# Antifungal effect of 4-arylthiosemicarbazides against *Candida* species. Search for molecular basis of antifungal activity of thiosemicarbazide derivatives

**DOI:** 10.1007/s00894-012-1420-5

**Published:** 2012-04-26

**Authors:** Agata Siwek, Joanna Stefańska, Katarzyna Dzitko, Artur Ruszczak

**Affiliations:** 1 Department of Organic Chemistry, Faculty of Pharmacy, Medical University, Chodźki 4a, 20-093 Lublin, Poland; 2 Department of Pharmaceutical Microbiology, Medical University, Oczki 3, 02-007 Warszawa, Poland; 3 Department of Immunoparasitology, University of Lodz, Banacha 12/16, 90-237 Łódź, Poland

**Keywords:** Thiosemicarbazide derivative, Antifungal activity, Cytotoxicity, Structure-activity relationship, Molecular docking

## Abstract

The in vitro antifungal potency of six series of 4-arylthiosemicarbazides was evaluated. Two isoquinoline derivatives with an ortho-methoxy or ortho-methyl group at the phenyl ring were the most potent antifungal agents. Molecular modeling studies and docking of all 4-arylthiosemicarbazides into the active sites of sterol 14α-demethylase (CYP51), topoisomerase II (topo II), l-glutamine: d-fructose-6-phosphate amidotransferase (GlcN-6-P), secreted aspartic proteinase (SAP), *N*-myristoyltransferase (NMT), and UDP-N-acetylmuramoyl-l-alanine:d-glutamate ligase (MurD) indicated the importance of both structural and electronic factors in ligand recognition and thus for the antifungal effectiveness of 4-arylthiosemicarbazides. A possible antifungal target was identified (NMT) and isoquinoline-thiosemicarbazides showed more favorable affinity than the native ligand.

**Electronic supplementary material:**

The online version of this article (doi:10.1007/s00894-012-1420-5) contains supplementary material, which is available to authorized users.

**Figure:**
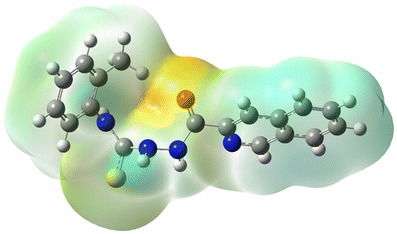
Electrostatic potential surface of isoquiniline derivative compound 6o with antifungal activity

## Introduction

Invasive fungal infections (IFIs) are life-threatening opportunistic infections that are an increasingly important cause of morbidity and mortality in patients, especially those with compromised immune function and those hospitalized with serious underlying diseases [1, 2]. The majority of these infections are caused by *Candida* spp., with over 50 % due to *Candida albicans* [3–6]. *Candida* species are the fourth most common pathogens isolated from patients with nosocomial bloodstream infections in the United States and the sixth in Europe [7–9]. These fungi are responsible for various forms of disease, ranging from superficial infections of the mucosal surfaces or skin to systemic infections, which in most cases are life threatening [10]. The incidence of candidemia in the US and Europe varies between 1.9 and 11 per 100,000 inhabitants [11–13]. Mortality in patients with candidemia is high, ranging from 40 % to 60 %, with reported attributable mortality of 20–40 % [9]. In general, for treatment of an infection with *Candida* species, amphotericin B and azole drugs are used, but these agents are not considered to satisfy medical needs because of their toxicity, side effects, drug interactions, limited routes, and the emergence of drug-resistant and drug-low-susceptible strains [14–21]. Among these limitations, the major obstacle in the treatment of *C. albicans* infections is the spread of antifungal drug resistance, mainly in patients chronically subjected to antimycotic therapy, i.e., those treated with broad-spectrum antibiotics, immunosuppressive agents, anticancer, and anti-AIDS drugs [22, 23]. Considering all these factors, the identification of new antifungal small molecules is an important goal of current anti-infective research.

Recently, as a part of our efforts to develop new effective antibacterial agents in the class of thiosemicarbazide derivatives, a series of 4-arylthiosemicarbazides was synthesized and their biological potency evaluated [24]. In vitro antibacterial activity assays indicated that compounds with electron-withdrawing substituents in the para position are more effective. Furthermore, it was documented for the first time that thiosemicarbazide derivatives participate in at least two different mechanisms of antibacterial activity. One of these was identified as inhibition of topoisomerase IV, while the nature of the other could not be elucidated from the limited data collected. The binding mode of the synthesized compounds was explored by flexible molecular docking, which indicated the importance of H-bonding and electrostatic interactions between the thiosemicarbazide core and amino acid residues of the ATP binding site. To further explore the diverse biological activity of thiosemicarbazide derivatives, we focused our attention on the antifungal activity and structure-activity relationships (SAR) of the 4-arylthiosemicarbazides, those already described [24–27] and nine new derivatives, using yeast *Candida* as the experimental model. Since selective toxicity is fundamental to the development of anti-infective agents, cytotoxicity studies were also carried out. Although the antifungal potential of thiosemicarbazide derivatives is well-recognized [28–37], thus far no detailed studies have been conducted to determine the mechanism of action and the target proteins for their antifungal activity. As most of the existing fungal drugs are enzyme inhibitors, the second aim of the present studies was to identify the interactions of 4-arylthiosemicarbazides with antifungal drug target enzymes using in silico molecular docking. In these studies, six common and novel enzymes that were considered in antifungal studies reported in literature [38–41], were selected as targets, i.e., sterol 14α-demethylase (CYP51), topoisomerase II (Topo II), l-glutamine:  d-fructose-6-phosphate amidotransferase (GlcN-6-P), secreted aspartic proteinase (SAP), *N*-myristoyltransferase (NMT), and UDP-N-acetylmuramoyl- l-alanine: d-glutamate ligase (MurD).

## Materials and methods

### Chemicals

All commercial reactants and solvents were purchased from either Sigma-Aldrich (St. Louis, MO) or Lancaster (Windham, NH) with the highest purity and used without further purification. Melting points were determined on a Fischer-Johns block and are uncorrected. Elemental analyses were determined by a AMZ-CHX elemental analyzer (within ± 0.4 % of theoretical values). IR spectra were recorded in KBr using a Specord IR-75 spectrophotometer. ^1^H NMR spectra were recorded on a Bruker Avance (300 MHz). Analytical thin layer chromatography (TLC) was performed on Merck 60F_254_ silica gel plates and visualized by UV irradiation (254 nm).

### Procedure for synthesis of 4-arylthiosemicarbazides

A reaction mixture of appropriate heterocarboxylic hydrazide (0.01 mol) and related isothiocyanate (0.01 mol) was heated in an oil bath at 80 °C and the progress of the reaction was monitored by TLC. After 12 h, the reaction was completed and crude reaction mixture was washed with diethyl ether and crystallized from ethanol.

Physicochemical characterization of 1a-1 m, 3d, 3n, 4b, 4d, 4p, 4q, 5c, 5h, 5i, 5m, 5n, and 6b were presented previously [24–27]. Compounds 2c, 2h, 2m, 2n are commercially available.

### Procedure for synthesis of *s*-triazole 6o-t

The thiosemicarbazide derivative 6o (0.01 mol) was dissolved in 2 % NaOH (10 mL) and refluxed for 2 h. After cooling, the solution was neutralized with 3M HCl. The solid formed was filtered, dried and crystallized from ethanol.

Yields and spectral characterization of new compounds are provided in Supplementary Material.

### Antifungal assay

The primary screen was carried out by the disc-diffusion method using agar medium, according to the Clinical and Laboratory Standards Institute guidelines [42]. For compounds showing an inhibitory effect on the growth of the tested microorganisms—monitored as an appearance of growth inhibition zones (GIZs)—minimal inhibitory concentrations (MICs) were determined using the agar dilution method, according to Clinical and Laboratory Standards Institute guidelines [43]. The detailed procedure was described in a previous paper [31].

### Cytotoxicity assay

#### Cell culture

Cell line L929 (ATTC^®^ Catalog No. CCL-1, mouse fibroblasts; http://www.atcc.org) were routinely cultured in Iscove’s modified Dulbecco medium (IMDM, Cytogen, Princeton, NJ), supplemented with 10 % (v/v) fetal bovine serum (FBS, Sigma), plus 2 mM l-glutamine (Sigma), 100.0 U/ml penicillin (Sigma), 100.0 μg/ml streptomycin (Sigma), 5x10^−5^ M 2-mercaptoethanol (Sigma) and grown at 37 °C in a 10 % CO_2_ humidified environment.

Suspensions of the compounds 1f, 1h, 1m, 2h, 5h, 6b and 6o were freshly prepared before the cells were exposed, and diluted to appropriate concentrations; 1–1,250 μg/mL for 1f, 1h, 1m, 1–500 μg/mL for 2h, 5h, 6o, and 1–300 μg/mL for 6b with the culture medium (containing 2.5 % DMSO). Cells treated with 2.5 % DMSO-solvent served as a control in each experiment.

#### Cell viability assays

The effects of tested compounds on the viability of mouse fibroblasts L929 cells were evaluated using the MTT [3-(4,5-dimethylthiazol-2-yl)-2,5-diphenyltetrazolium bromide] assay. The MTT assay was used according to international standards: ISO 10993-5:2009 (Tests for in vitro cytotoxicity; http://www.iso.org/iso/catalogue_detail.htm?csnumber=36406 ). L929 cells were plated into 96-well plates at a density of 1.0 x 10^4^/100 μl/well in culture medium and allowed to attach for 24 h before treatment. Afterwards, culture medium in the plates was replaced by 100 μl compounds suspension at concentration of 0–565 μg/ml and the cells were exposed for 24 h. Then 1 mg/ml MTT (50 μl/well) was added to each well and incubated at 37 °C, 10 % CO_2_ for 2 h. Mitochondrial dehydrogenases of viable cells reduce the yellowish water-soluble MTT to water-insoluble formazan crystals, which were solubilized with dimethyl sulfoxide (DMSO). The cell culture medium was aspirated cautiously, after which 150 μl DMSO was added to each well and mixed thoroughly. Optical density (OD) was read on the ELISA reader (Multiskan EX, Labsystems; http://www.mtxlsi.com/multiskan_EX.htm) at 550 nm. The results were expressed as percentage viability compared with the treated 2.5 % DMSO controls. All experiments were performed in triplicate.

### Computational details

Physicochemical parameters, HOMO/LUMO maps, and conformational searches were calculated using HyperChem8.0.3 [44]. Extensive conformational searches were carried out using the molecular mechanics level with the OPLS [45, 46] force field. The most stable structures obtained were subsequently optimized to the closest local minimum at the semiempirical level using RM1 parametrization [47]. Convergence criteria were set to 0.1 and 0.01 kcal mol^−1^ Å^−1^ for OPLS and RM1 calculations, respectively. Electrostatic potentials were calculated for the geometries that resulted from docking using Gaussian 03 [48] and GaussView 5 [49] at the HF/6-31G level [50, 51].

### Automated docking setup

Flexible ligand–receptor docking was performed using the AutodockVina program [52] using the default settings. Models of the sterol 14α-demethylase (CYP51), topoisomerase II (topo II), l-glutamine:  d-fructose-6-phosphate amidotransferase (GlcN-6-P), secreted aspartic proteinase (SAP), *N*-myristoyltransferase (NMT), and UDP-N-acetylmuramoyl- l-alanine: d-glutamate ligase (MurD) binding sites based on the structure deposited in the Protein Data Bank [53] under the PDB ID 2CIB [54], 1Q1D [55], 1XFF [56], 1EAG [57], 1IYL [58], 1UAG [59] were employed. Default docking parameters and flexible space of 24 x 24 x 24 Å^3^ were validated by re-docking native ligand that docked exactly in the position present in the crystal structure. Subsequently, all 4-arylthiosemicarbazides were docked using same docking parameters.

## Results and discussion

### Chemistry

**Scheme 1:**
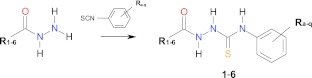
Preparation of targeted 4-arylthiosemicarbazides, series 1–6. Series 1: 1a–m; series 2: 2c, 2h, 2i, 2m, 2n; series 3: 3c, 3d, 3h, 3m, 3n, 3q; series 4: 4b, 4d, 4k, 4p, 4q;series 5: 5c, 5h, 5i, 5m, 5n; series 6: 6b, 6k, 6n, 6o, 6p. For symbols used to identify studied compounds see Table 1

Six series (1–6) of 4-arylthiosemicarbazides were prepared in high yields according to a known procedure [24–27, 60–63] in the reaction of related heterocarboxylic acid hydrazide with aryl isothiocyanate (Scheme 1). This one-step reaction produced nine new (2i, 3h, 3m, 3q, 4k, 6k, 6n, 6o, 6p) and 30 known thiosemicarbazides derivatives (1a-1m, 2c, 2h, 2m, 2n, 3c, 3d, 3n, 4b, 4d, 4p, 4q, 5c, 5h, 5i, 5m, 5n, 6b).

### In vitro antifungal activity

The in vitro antifungal activity of the title 4-arylthiosemicarbazides (series 1–6) was tested in comparison with fluconazole against three strains of yeast—*C. albicans* ATCC 10231, *C. albicans* ATCC 90028 and *C. parapsilosis* ATCC 22019—using the agar dilution method as described in CLSI documents M7–A7 [43]. Minimal inhibitory concentrations (MICs) were defined as the lowest concentration of the compound preventing growth of the tested microorganism and are listed in Table 2. The results indicated that within series 1, compounds 1c with the para-nitro substitution, and 1f with the ortho and para positions substituted with chlorine atoms were the most potent, exhibiting moderate activity of 50 μg/mL towards *C. parapsilosis.* These compounds were also effective against *C. albicans*, however at much higher concentrations (MIC range 100–200 μg/mL). Two other derivatives; 1h with para-fluoro substitution and 1m with ortho-bromo substitution showed somewhat lower activity, whereas remaining compounds with halo substitution at the ortho and/or para position, 2,4-difluoro derivative 1i, para-bromo derivative 1k, and para-chloro derivative 1l, were inactive. When the thiadiazole scaffold was replaced with the similarly sized thiophene, pyrrole or furane (series 2, 3 and 4, respectively) antifungal potency was lost completely except at 2h. Subsequently, the antifungal potency of series 5 with an indole scaffold was studied. This search led to the identification of para-fluoro derivative 5h and 2,4-difluoro derivative 5i with MIC at 100 μg/mL towards *C. parapsilosis* and marginal activity towards *C. albicans*. Remaining derivatives with ortho-fluoro 5n, para-nitro 5c, and ortho-bromo 5m substitution were inactive. Replacement of the indole scaffold with the similarly sized isoquinoline resulted in ortho-methoxy derivative 6o with MIC at 25 μg/mL towards *C. albicans* and 50 μg/mL towards *C. parapsilosis*. An antifungal response was also observed for ortho-methyl derivative 6b, however, at somewhat higher concentration; MICs at 50 μg/mL towards all screened *Candida* species. Interestingly, derivatives with electron-withdrawing substitution, para-bromo derivative 6k, ortho-fluoro derivative 6n, and para-iodo derivative 6p were inactive. Finally, the biological activity of *s*-triazole 6o-t, a cyclic derivative of the most potent 6o, was tested. No antifungal potency was noted.

**Table 1:**
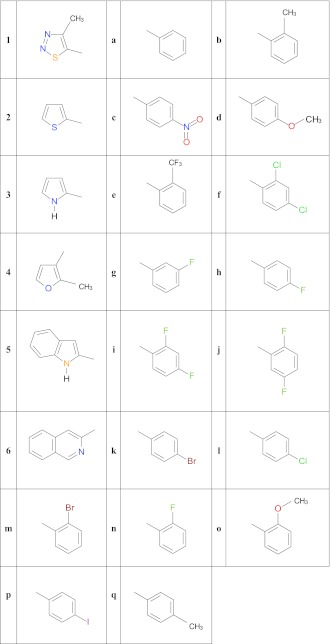
Symbols used to identify studied compounds using substituents at thiosemicarbazide moiety

**Table 2:** In vitro antifungal activity [minimal inhibitory concentration (MIC; μg/mL) of 4-arylthiosemicarbazides]

Compound	*Candida albicans* ATCC 10231	*C. albicans* ATCC 90028	*Candida parapsilosis* ATCC 22019
1c	100	200	50
1f	200	200	50
1 h	>400	400	100
1 m	400	200	100
2 h	400	200	50
5 h	>400	>400	100
5i	>400	400	100
6b	50	50	50
6o	25	25	50
fluconazole	1	1	2

### Cytotoxicity of selected compounds against L929 cells

Compounds 1f, 1h, 1m, 2h, 5h, 6b, and 6o, all of which showed in vitro antifungal activity, were further examined for their initial in vitro toxicity effects against mammalian L929 cells by MTT assay. Toxicity was defined as the highest dilution of test samples that causes 30 % or greater destruction of cells. Morphology of normal cells and cultured cells with tested compounds are given in Fig. S1 in Supplementary Material.

**Fig. 1:**
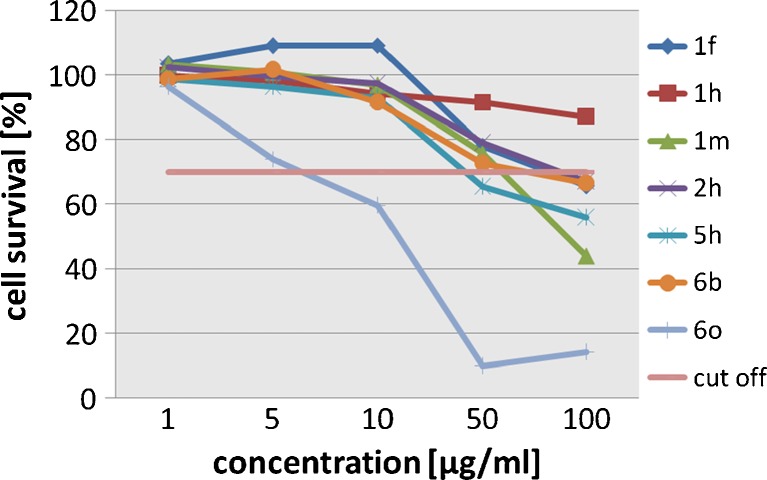
Concentration-dependent cytotoxic activity of 1f, 1h, 1m, 2h, 5h, 6b, and 6o

From a pharmacological point of view it is important for the studied compounds to exhibit high bioactivity and at the same time show no or low toxicity effects, otherwise the activity might just be due to general toxicity, which disqualifies the compound as a drug or lead molecule candidate. From a comparison of the results of toxicity collected in Fig. 1 and antifungal activity tests, it can be seen clearly that the potent anti-*Candida* agent 6b displays antifungal activity at non-cytotoxic concentrations in mammalian cells. Also, compounds 1f, 1h, and 2h were found to be non-toxic up to 50 and 100 μg/mL, respectively, which equals the MIC values against *C. parapsilosis*. Another important result from the toxicity studies is that the toxicity of the tested compounds does not seem to be correlated to their antifungal activity. For example, 1f, which was found to be non-toxic up to the same concentration as 6b (50 μg/mL) is four-time less active against *Candida* strains (MIC of 200 vs 50 μg/mL). Compound 1h, which was non-toxic up to 100 μg/mL, is only marginally active (MIC 400 μg/mL or higher against *Candida* strains). In contrast, compound 5h, which is even less antifungal than 1h was found to be non-toxic up to 10 μg/mL, while the most cytotoxic 6o (5 μg/mL) is the most potent antifungal. This indicates either that these compounds may act via different mechanisms in their antifungal activity against *C. albicans* and toxicity against mammalian cells or that minor structural changes afford important alterations in fungal cell permeability/transport, etc. [64]

### Molecular modeling studies

**Fig. 2:**
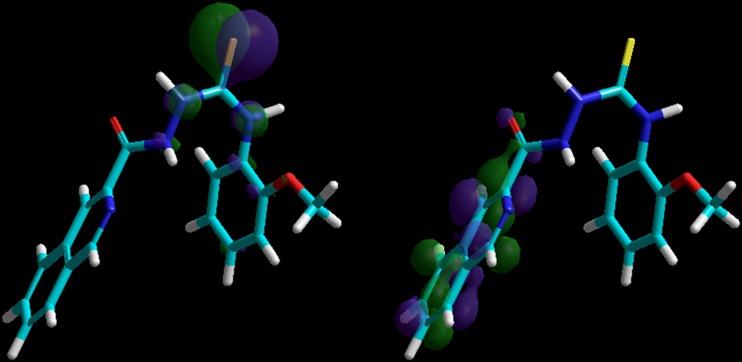
Highest occupied molecular orbital (HOMO;* left*) and lowest unoccupied molecular orbital (LUMO;* right*) contour plots for representative model compound 6o. HOMO and LUMO maps for representative model compounds series 1 and 5 are presented in our previous paper [24]

Since the in vitro antifungal activity could not be explained reasonably in the light of theories of the influence of substituents on biological response, the difference in bioactivity of 4-arylthiosemicarbazides was therefore investigated further by a molecular modeling approach and docking studies with the hope of finding a possible explanation for the observations described above, and to provide a better understanding of the relationship between structure and antifungal activity. Initially, some structural and electronic descriptors of the title compounds were investigated (see Table S1 in the Supplementary Material). In the structure of 4-arylthiosemicarbazide, the presence of the isoquinoline ring and electron-donating methyl or methoxy groups (compounds 6b and 6o) seemed to confer higher antifungal potency than that of other substituted groups. They also are characterized by favorable *E*_B_ (binding energy) values and high *μ* (dipole moment) and *E*_HOMO_ (the highest occupied molecular orbital energy) values in contrast to all other studied compounds. Antifungal activity is probably affected by these electronic descriptors, which can be important parameters for the interaction of 4-arylthiosemicarbazides with the active sites. It is known that the energies of HOMO and lowest unoccupied molecular orbital (LUMO) orbitals serve as indices for the electron-donating and electron-acceptor abilities of the molecule, respectively. The higher the energy of the HOMO, the better its electron-donating ability. In the case of LUMO, good electron-acceptor ability is associated with low-energy molecular orbitals. In our results, antifungal potency is related to HOMO, which suggests that electron-donor ability of the molecule is important in determining activity. According to the frontier orbital maps presented in Fig. 2 for representative model compound 6o, the HOMOs of each compound were localized mainly on the sulfur atom of the thiosemicarbazide core. These observations emphasize the possible role of the sulfur atom in the charge transfer processes that occur when a ligand interacts with the biological target.

Subsequently, the localization of LUMO orbitals for each structure was analyzed. There was no quantitative correlation between antifungal activity and LUMO localization, but a trend clearly emerged; 4-arylthiosemicarbazides from series 1, 2, 5, and 6 have their LUMO situated on the heterocyclic ring, whereas LUMO density for compounds from completely inactive series 3 and 4 was distributed outside the heterocyclic moiety, mainly on the sulfur atom. This behavior might be interpreted in the sense that 4-arylthiosemicarbazides with the electron-accepting character of the heterocyclic ring at N1 position interact better with the biological target.

Finally, conformational analysis of the studied compounds was performed according to the protocol described for 4-arylthiosemicarbazides with thiadiazole (series 1) and indole (series 5) moieties [24]. As expected based on our previous studies, no structural requirements needed for antifungal activity of 4-arylthiosemicarbazides could be deduced; both isoquinoline derivatives with significant activity, 6b and 6o, as well as inactive ones 6k, 6n, and 6p shared very similar geometry. Again, comparison of the molecular shape of active thiophene derivative 2h with the most potent antifungal 6o or inactive ones 2m, 2n, 3h, 3d, 4b did not show any recognizable relationship with activity (data not shown). Thus, the present study manifested the importance of electronic properties rather than the restricted stereochemistry of the molecule for the antifungal response.

### Docking studies

**Fig. 3:**
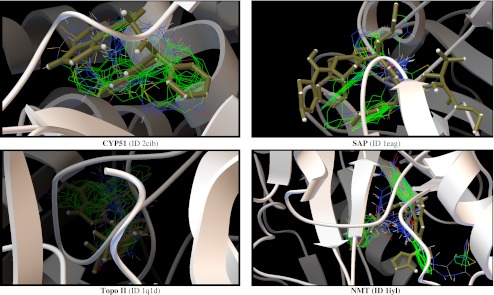
Superimposition of the native ligand (rendered as* tubes*) and the best conformations of representative ligands 1a, 1b, 1c, 1f, 1 h, 1 m, 2c, 2 h, 3n, 3q, 4d, 4 k, 5c, 5 h, 5i, 5n, 6b, 6n, 6o, and 6p docked to the binding site of sterol 14α-demethylase (CYP51) (ID 2cib), topoisomerase II (Topo II) (ID 1q1d), secreted aspartic proteinase (SAP) (ID 1eag), and *N*-myristoyltransferase (NMT) (ID 1iyl)

As noted in the Introduction, with the hope if identifying the fungal cellular targets of thiosemicarbazide class compounds, we analyzed interactions of active and inactive molecules with the active sites of common and novel enzymes that were considered in antifungal studies reported in the literature [38–41]. The following enzymes were included in the studies: sterol 14α-demethylase (CYP51), topoisomerase II (Topo II), l-glutamine:  d-fructose-6-phosphate amidotransferase (GlcN-6-P), secreted aspartic proteinase (SAP), *N*-myristoyltransferase (NMT), and UDP-N-acetylmuramoyl- l-alanine: d-glutamate ligase (MurD). The docking simulations were performed using the program AutoDock Vina, which was applied successfully in our previous studies of the binding mode of thiosemicarbazide derivatives in the active site of bacterial topoisomerase II [24]. The best poses of the studied compounds within the active sites of target enzymes are displayed in Fig. 3 and their corresponding binding Gibbs free energies ΔG_b_ are shown in Table 3.

**Table 3:** Binding free energy (ΔG_b_) corresponding to the best docking poses of compounds from series 1–6 in the active site of CYP51 (ID 2cib), Topo II (ID 1q1d), GlcN-6-P (ID 1xff), SAP (ID 1eag), NMT (ID 1iyl), and MurD (ID1uag)

Compound	ΔG_b_ (kcal/mol)					
	CYP51	Topo II	GlcN-6-P	SAP	NMT	MurD
Ligand	-11.5	-11.8	-7.9	-8.0	-9.1	-5.1
1a	-6.8	-8.4	2.9	-7.1	-7.4	19.2
1b	-7.1	-8.4	5.0	-7.0	-8.1	22.8
1c	-7.1	-8.6	4.6	-7.0	-7.6	22.8
1d	-7.0	-8.4	5.2	-7.1	-7.1	20.8
1e	-7.5	-8.9	7.3	-7.4	-8.4	24.8
1f	-7.1	-8.9	7.8	-7.4	-7.6	24.9
1 g	-6.9	-8.6	3.3	-7.2	-7.6	19.9
1 h	-7.0	-8.2	3.4	-7.2	-7.8	19.4
1i	-7.3	-8.9	4.9	-7.7	-7.8	20.8
1j	-7.2	-8.8	4.8	-7.8	-7.7	21.6
1 k	-7.1	-8.5	7.8	-6.9	-7.2	21.7
1 l	-7.1	-8.5	5.3	-7.3	-7.3	21.2
1 m	-7.2	-8.3	5.9	-6.7	-7.6	23.6
2c	-7.1	-8.2	7.1	-6.8	-7.6	20.8
2 h	-7.5	-7.7	2.5	-6.7	-7.6	19.2
2i	-7.4	-7.9	2.1	-7.4	-7.8	19.1
2 m	-7.1	-7.7	3.8	-6.6	-7.4	21.2
2n	-7.2	-7.7	1.8	-7.2	-7.7	18.6
3c	-7.3	-8.7	2.4	-6.5	-7.7	18.6
3d	-7.5	-8.0	2.6	-6.6	-7.5	17.6
3 h	-7.7	-8.1	1.7	-7.3	-7.6	14.8
3 m	-7.4	-7.9	2.6	-6.3	-7.8	18.4
3n	-7.0	-8.1	0.9	-7.5	-7.9	15.6
3q	-7.8	-8.1	1.8	-6.9	-7.7	16.4
4b	-7.5	-8.0	4.9	-7.4	-8.1	20.6
4d	-7.1	-7.8	5.7	-7.0	-7.4	21.1
4 k	-7.3	-7.8	7.0	-7.0	-7.4	22.1
4p	-7.2	-7.9	8.0	-7.0	-7.4	24.5
4q	-7.5	-8.0	3.6	-7.3	-7.5	19.5
5c	-9.7	-10.0	12.2	-7.2	-8.2	34.1
5 h	-9.4	-8.4	10.6	-7.5	-8.2	28.4
5i	-9.6	-8.7	11.6	-7.8	-8.7	30.9
5 m	-9.5	-9.1	10.6	-8.8	-8.1	32.5
5n	-9.4	-8.6	8.0	-7.5	-7.7	29.1
6b	-7.9	-8.8	11.9	-7.0	-10.1	33.6
6 k	-8.2	-9.1	17.2	-7.0	-10.1	37.9
6n	-7.9	-8.9	11.7	-7.4	-9.8	31.2
6o	-7.7	-9.0	18.5	-6.9	-9.5	34.6
6p	-8.2	-8.9	19.3	-7.0	-10.0	40.6

**Fig. 4:**
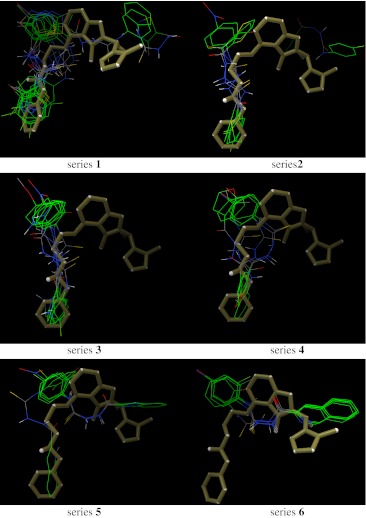
Superimposition of the native ligand (rendered as* tubes*) and the best conformations of ligands from series 1–6 docked to the binding site of NMT (1iyl)

As indicated by the positive value of the Gibbs free energy, none of the ligands bound the active sites of GlcN-6-P and MurD, while potential inhibitory activities of the studied compounds against CYP51, Topo II, SAP, and NMT were observed. Almost all best poses in the series 1–6 were located in a position very similar to that of the inhibitors on which these enzymes were crystallized. Some SAR trends were observed when the docking conformations and binding free energies of the studied compounds in the active site of NMT were analyzed. The nonpeptidic inhibitor that NMT was crystallized with contains a benzofuran core and a long aminoaliphatic chain. From among the compounds studied by us, those containing an isoquinoline ring (series 6) show more favorable binding affinity than the native ligand, and bind to the site occupied by the benzofuran ring. The remaining compounds, series 1–5, show binding Gibbs free energies lower than the native ligand, and bind to the site occupied by the aminoaliphatic chain situated at position 4 of the benzofuran ring, except for 1a, 1b, 2h, 5c, 5h, 5i, and 5m (Fig. 4). This supports the conclusion reached from experiments [58] that the part of the active site to which the benzofuran moiety is allocated is responsible mainly for binding. Another significant finding is that, with the exception of 2h and 2i, all compounds bind to the active site of NMT without H-bond interactions with amino acid residues. This is a very important result, which points to the fact that the electronic nature of a molecule may play the key role in ligand recognition.

**Fig. 5:**
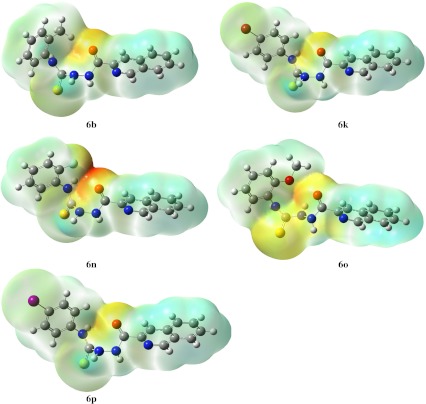
Comparison of the electrostatic potential surfaces of compounds from series 6 that resulted from docking studies at the active site of NMT

Subsequently, we focused on isoquinoline derivatives (series 6) as this series contains the two most active compounds: 6b and 6o. Based on the conformations obtained from docking studies, we generated electrostatic potential surfaces. As seen in Fig. 5, the most active compound (6o) is characterized by electrostatics around the sulfur atom that differ significantly from that in the other ligands. The is explained mainly by the opposite orientation of the proton in the neighboring NH group. Following this finding, we analyzed the geometry of this fragment of the thiosemicarbazide skeleton in detail (Table 4). The opposite location of the NH proton is characterized by the HN(N)CS dihedral angle being significantly different, and opposite in sign to that in all other compounds. The graphical representation of the different electrostatics in this part of 6o presented in Fig. 5 was quantified by the Mulliken partial atomic charge at the sulfur atom, which was also significantly different and opposite in sign than in other ligands. Two other geometric parameters, i.e., C–S bond distance and HN(C)CS dihedral angle, are also distinct in the case of 6o. It should be noted that these differences are very subtle and can be easily overlooked. The importance of electrostatics and geometry of the –NH–C(=S)–NH– fragment can be further inferred from the corresponding parameters of the methyl derivative 6b. As can be seen in Table 4, 6b is closer to 6o than to the remaining ligands. This correlates well with bioactivity, with that of 6b being lower than that of 6o and the other ligands being inactive.

**Table 4:** Sulfur charge density (|e^−^|), selected bond lengths (Å) and torsional angles (°) of compounds from series 6

Compound	S charge density	C = S	HN(C)CS	HN(N)CS
6b	0.012	1.610	-58.8	-71.0
6 k	0.064	1.608	-79.6	-58.9
6n	0.074	1.608	-87.1	-56.1
6o	-0.016	1.612	-99.8	171.6
6p	0.070	1.609	-82.1	-60.9

**Fig. 6:**
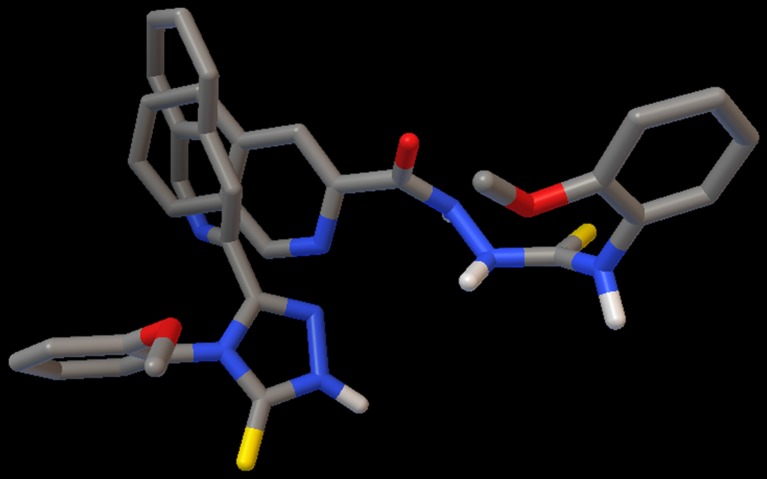
Superimposition of the best conformations of 6o and 6o-t docked to the binding site of NMT (1iyl)

Based on the above analysis, we also addressed the question of the lack of antifungal activity of *s*-triazole 6o-t, which is a dehydroderivative of the most active thiosemicarbazide 6o. We docked compound 6o-t to the active site of NMT. As illustrated in Fig. 6, the best pose of 6o-t shows only partial overlap with the corresponding pose of 6o, which coincides well with the position of the ligand used in crystallographic studies. Most importantly, the orientation of the N–C(=S)–N fragment of the *s*-triazole ring is completely different and the location of the sulfur atom within the active site is completely different, 8.7 Å away from the location of sulfur atom of 6o, which, as argued above, influences the antifungal activity of this ligand. Thus, the structural and electronic factors of the NH-NH–C(=S)–NH core seem to be important for ligand recognition and, consequently, for the antifungal effectiveness of the studied 4-arylthiosemicarbazides. Only use of specific enzymatic bioassays, will allow this hypothesis to be confirmed and the biological target of the tiosemicarbazide class of compounds to be identified unequivocally.

## Conclusions

The present study evaluated the in vitro antifungal activities of six series of 4-arylthiosemicarbazides. Among the compounds tested, two isoquinoline derivatives 6o and 6b were the most potent antifungals. Subsequently, molecular modeling and flexible docking studies were carried out to identify and characterize the structural and electronic properties that modulate the antifungal potency of 4-arylthiosemicarbazides. The electronic requirements for antifungal effectiveness deduced from the molecular modeling approach are (1) high HOMO and dipole moment values, (2) favorable binding energy, and (3) the presence of an electron accepting heteroaromatic ring at N1 position of thiosemicarbazide skeleton. The structural and electronic requirements for ligand recognition deduced from comparisons of ligands in the active site of NMT are (1) high-electron density around the sulfur atom, and (2) geometry of NH-NH-C(=S)-NH core. The results of the docking study of inactive *s*-triazoles 6o-t, a dehydroderivative of the most potent antifungal agent 6o, confirmed the validity of the pharmacophore model developed for 4-arylthiosemicarbazides, although additional experimental evidence is needed to support this hypothesis. Efforts aimed at elucidating the antifungal mechanism of action of 4-arylthiosemicarbazides using biochemical approaches are underway in our group.

## Supplementary material available

Yields and spectral characterization of new compounds. Figure S1 shows morphology of normal cells and cultured cells with 1f,1 h, 1 m, 2 h, 5 h, 6b, and 6o, and Table S1 gives calculated molecular descriptors of thiosemicarbazide derivatives 1–6.

ESM 1 (DOC 4706 kb)
