# Supplementary material for: Antifungal effect of 4-arylthiosemicarbazides against Candida species. Search for molecular basis of antifungal activity of thiosemicarbazide derivatives
Source: J Mol Model. 2012 Apr 26;18(9):4159–70. doi: 10.1007/s00894-012-1420-5 (PMC3429777; doi:10.1007/s00894-012-1420-5)
Supplement: Supplementary file 1 — (DOC 4706 kb) [file 894_2012_1420_MOESM1_ESM.doc]

**Supplementary data**

**Antifungal effect of 4-arylthiosemicarbazides against *Candida* species.**

**Search for molecular basis of antifungal activity of thiosemicarbazide derivatives**

Agata Siwek,a,[[1]](#footnote-2)* Joanna Stefańska,^b^ Katarzyna Dzitko,^c^ Artur Ruszczak^c^

*^a^ Department of Organic Chemistry, Faculty of Pharmacy, Medical University, Chodźki 4a, 20-093 Lublin, Poland*

*^b^ Department of Pharmaceutical Microbiology, Medical University, Oczki 3, 02-007 Warszawa, Poland*

*^c^ Department of Immunoparasitology, University of Lodz, Banacha 12/16, 90-237 Łódź, Poland*

Contents

Title page S1

1. Physicochemical characterization of new compounds S2-S3

2. Cytotoxic activity of **1f**, **1h**, **1m**, **2h**, **5h**, **6b**, and **6o** (Fig. S1) S3

3. SAR parameters of 4-arylthiosemicarbazides **1-6** (Table S1) S4-S7

**1. Physicochemical characterization of new compounds**

4-(2,4-difluorophenyl)-1-(thiophen-2-yl)-carbonylthiosemicarbazide **2i**

Yield: (2.85 g, 91%). Mp: 195-7 ^o^C. IR (ν, cm^-1^) 3320 (NH), 1665 (C=O), 3035, 1620, 1583 (Ar-H), 1280 (C=S), 771 (C–S). ^1^H-NMR (DMSO-d_6_) δ_H_ 7.04-7.10 (m, 1H, CH), 7.19-7.31 (m, 3H, 3×CH), 7.85-7.87 (d, 2H, 2×CH), 9.65, 9.94, 10.64 (3s, 3H, 3×NH). Anal. C_12_H_9_F_2_N_3_OS_2_ (C, H, N).

4-(4-fluorophenyl)-1-(pyrrol-2-yl)-carbonylthiosemicarbazide **3h**

Yield: (2.47 g, 89%). Mp: 191-3 ^o^C. IR (ν, cm^-1^) 3311 (NH), 3098, 1623, 1590, 1510 (Ar-H.), 1645 (C=O), 1340 (C=S). ^1^H-NMR (DMSO-d_6_) δ_H_ 6.12-6.14 (m, 1H, CH), 6.84-6.90 (m, 2H, 2×CH), 7.08-7.12 (m, 2H, 2×CH), 7.40-7.46 (m, 2H, 2×CH), 9.63, 9.77, 10.01, 11.61 (4s, 4H, 4×NH). Anal. C_12_H_11_FN_4_OS (C, H, N).

4-(2-bromophenyl)-1-(pyrrol-2-yl)-carbonylthiosemicarbazide **3m**

Yield: (3.21 g, 95%). Mp: 195-7 ^o^C. IR (ν, cm^-1^) 3333 (NH), 3078, 1626, 1509, 1470 (Ar-H), 1607 (C=O), 1327 (C=S). ^1^H-NMR (DMSO-d_6_) δ_H_ 6.11-6.14 (m, 1H, CH), 6.94 (s, 2×H, 2CH), 7.13-7.61 (m, 4H, 4×CH), 9.52, 9.77, 10.10, 11.64 (4s, 4H, 4×NH). Anal. C_12_H_11_BrN_4_OS (C, H, N).

4-(4-methylphenyl)-1-(pyrrol-2-yl)-carbonylthiosemicarbazide **3q**

Yield: (2.38 g, 87%). Mp: 223-5 ^o^C. IR (ν, cm^-1^) 3302, 3201 (NH), 3123, 1574, 801 (Ar-H), 2941, 2839, 1435 (Aliph.), 1619 (C=O), 1344 (C=S). ^1^H-NMR (DMSO-d_6_) δ_H_ 2.41 (s, 3H, 3×CH), 5.22 (m, 1H, CH), 5.90 (m, 1H, CH), 6.91 (m, 1H, CH), 7.22-7.32 (m, 2H, 2×CH), 7.40 (m, 2H, 2×CH), 9.23, 9.69, 10.01, 11.63 (4s, 4H, 4×NH). Anal. C_13_H_14_N_4_OS (C, H, N).

4-(4-bromophenyl)-1-(2-methyl-furan-3-yl)-carbonylthiosemicarbazide **4k**

Yield: (3.19 g, 90%). Mp: 194-6 ^o^C. IR (ν, cm^-1^) 3360, 3258 (NH), 3166, 1603, 830 (Ar-H), 2965, 1488 (Aliph.), 1681 (C=O), 1357 (C=S), 1181 (C-O). ^1^H-NMR (DMSO-d_6_) δ_H_ 2.53 (s, 3H, 3×CH), 6.92 (d, 1H, CH), 7.43-7.52 (m, 4H, 4×CH), 7.56-7.57 (d, 1H, CH), 9.72, 9.80, 10.07, (3s, 3H, 3×NH). Anal. C_13_H_12_BrN_3_O_2_S (C, H, N).

4-(4-bromophenyl)-1-(isoquinolin-3-yl)-carbonylthiosemicarbazide **6k**

Yield: (3.68 g, 92%). Mp: 215-7 ^o^C. IR (ν, cm^-1^) 3292, 3223 (NH), 3122, 1598, 1588, 1500, 1477, 827 (Ar-H), 1655 (C=O), 1243 (C=S). ^1^H-NMR (DMSO-d_6_) δ_H_ 7.50 (s, 4H, 4×CH), 7.81-7.92 (m, 2H, 2×CH), 8.22-8.29 (dd, 2H, 2×CH), 8.63 (s, 1H, CH), 9.43 (s, 1H, CH), 9.82, 10.89 (2s, 3H, 3×NH). Anal. C_17_H_13_BrN_4_OS (C, H, N).

4-(2-fluorophenyl)-1-(isoquinolin-3-yl)-carbonylthiosemicarbazide **6n**

Yield: (3.03 g, 89%). Mp: 193-5 ^o^C. IR (ν, cm^-1^) 3294, 3202 (NH), 3094, 1616, 1601, 1498, 1475, 752 (Ar-H), 1658 (C=O), 1250 (C=S). ^1^H-NMR (DMSO-d_6_) δ_H_ 7.18-7.27 (m, 3H, 3×CH), 7.40 (s, 1H, CH), 7.81-7.92 (m, 2H, 2×CH), 8.22-8.29 (d, 2H, 2×CH), 8.64 (s, 1H, CH), 9.43 (s, 1H, CH), 9.57, 9.94, 10.94 (3s, 3H, 3×NH). Anal. C_17_H_13_FN_4_OS (C, H, N).

1-(isoquinolin-3-ylcarbonyl)-4-(2-methoxyphenyl)-thiosemicarbazide **6o**

Yield: (3.31 g, 94%). Mp: 210-12 ^o^C. IR (ν, cm^-1^) 3278, 3205 (NH), 3086, 3000, 1606, 1550, 1496, 1478, 751 (Ar-H), 2838, 1434 (Aliph.), 1650 (C=O), 1255 (C=S). ^1^H-NMR (DMSO-d_6_) δ_H_ 3.75 (s, 3H, 3×CH), 6.92-7.17 (m, 3H, 3×CH), 7.82-7.94 (m, 2H, 2×CH), 8.13-8.16 (d, 1H, CH), 8.23-8.30 (m, 2H, 2×CH), 8.65 (s, 1H, CH), 9.45 (s, 1H, CH), 9.20, 9.90, 11.08 (3s, 3H, 3×NH). Anal. C_18_H_16_N_4_O_2_S (C, H, N).

4-(4-iodophenyl)-1-(isoquinolin-3-yl)-carbonylthiosemicarbazide **6p**

Yield: (3.90 g, 87%). Mp: 216-8 ^o^C. IR (ν, cm^-1^) 3293, 3192 (NH), 3117, 1598, 1587, 1500, 1477, 826 (Ar-H), 1655 (C=O), 1243 (C=S). ^1^H-NMR (DMSO-d_6_) δ_H_ 7.34-7.37 (d, 2H, 2×CH), 7.64-7.67 (d, 2H, 2×CH), 7.81-7.92 (m, 2H, 2×CH), 8.22-8.29 (dd, 2H, 2×CH), 8.62 (s, 1H, CH), 9.43 (s, 1H, CH), 9.78, 9.80, 10.88 (3s, 3H, 3×NH). Anal. C_17_H_13_IN_4_OS (C, H, N).

3-(isoquinolin-3-yl)-4-(2-methoxyphenyl)-*s*-1,2,4-triazole-5-thione **6o**-**t**

Yield: (3.11 g, 93%). Mp: 251-3 ^o^C. IR (ν, cm^-1^) 3445 (NH), 3073, 1550, 1505, 751 (Ar-H), 2835, 1466, 1433 (Aliph.), 1258 (C=S). ^1^H-NMR (DMSO-d_6_) δ_H_ 3.46 (s, 3H, 3×CH), 7.04-7.09 (m, 2H, 2×CH), 7.38-7.46 (m, 2H, 2×CH), 7.71-7.77 (m, 1H, CH), 7.80-7.86 (m, 1H, CH), 8.05-8.10 (t, 2H, 2×CH), 8.30 (s, 1H, CH), 9.05 (s, 1H, CH), 14.19 (s, 1H, NH). Anal. C_18_H_14_N_4_OS (C, H, N).

**2. Cytotoxic activity of 1f, 1h, 1m, 2h, 5h, 6b, and 6o**

| 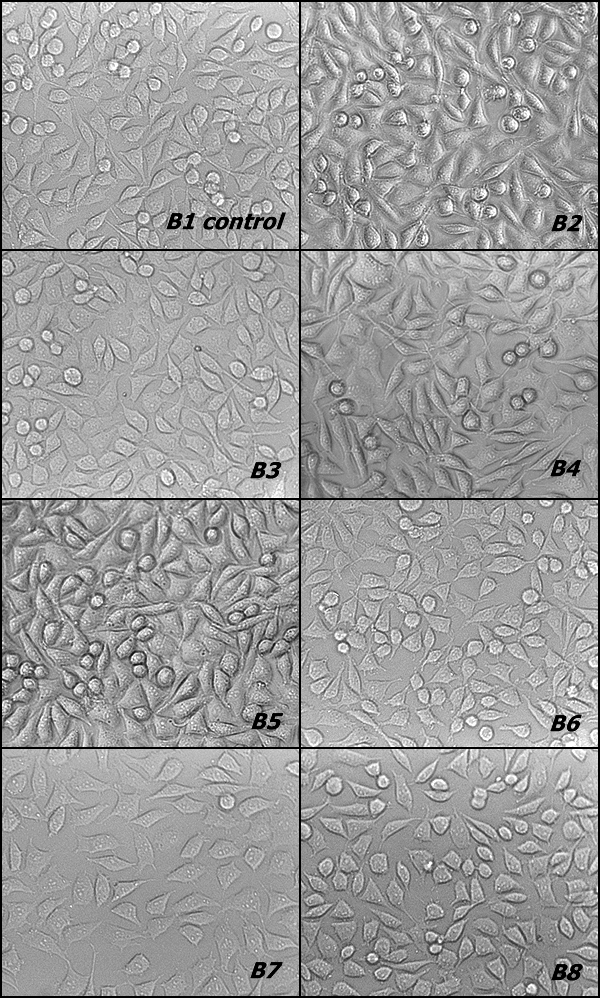 | 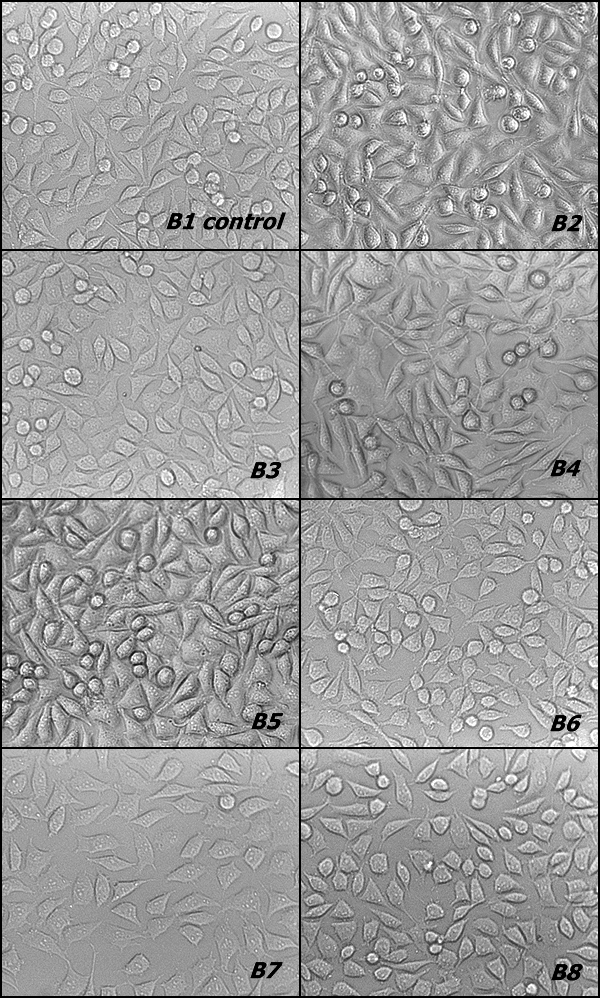 |
| --- | --- |
| **control** | **1f** |
| 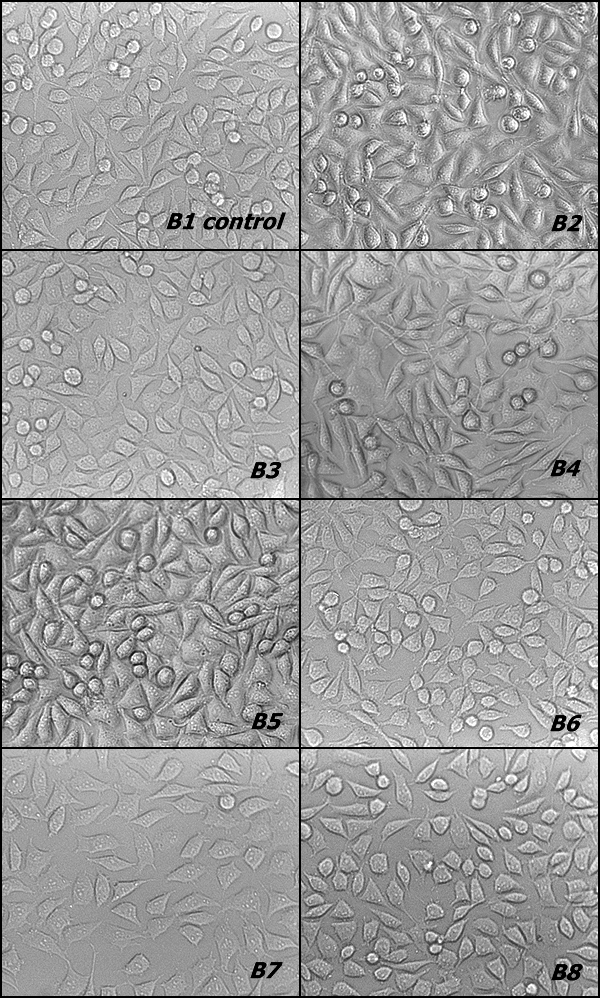 | 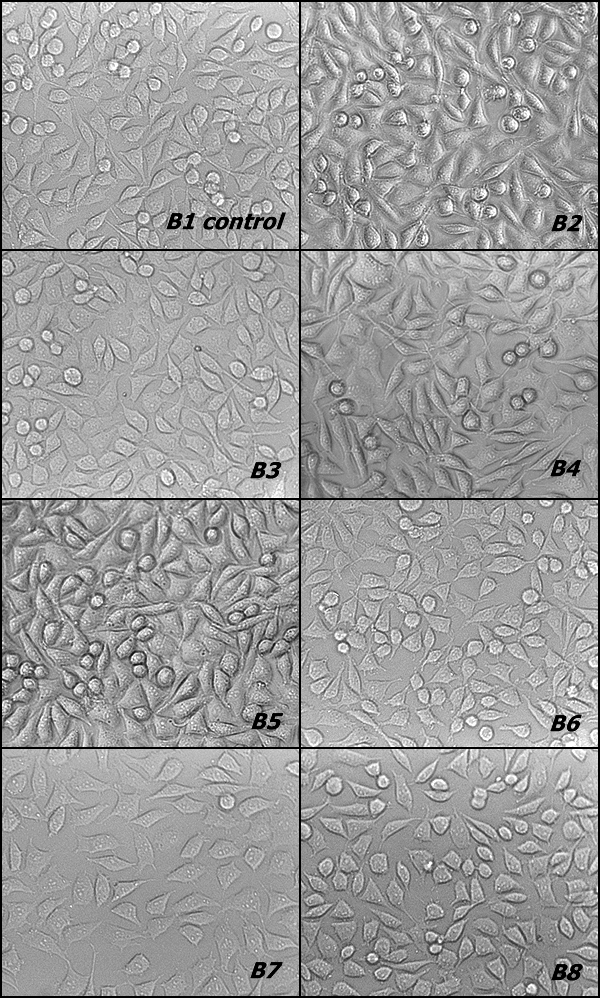 |
| **1h** | **1m** |
| 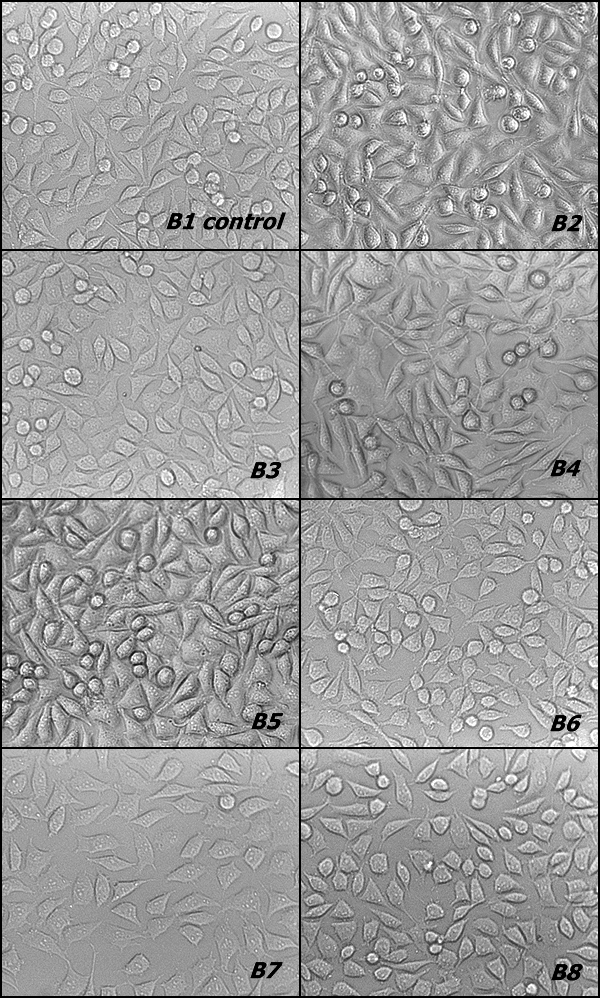 | 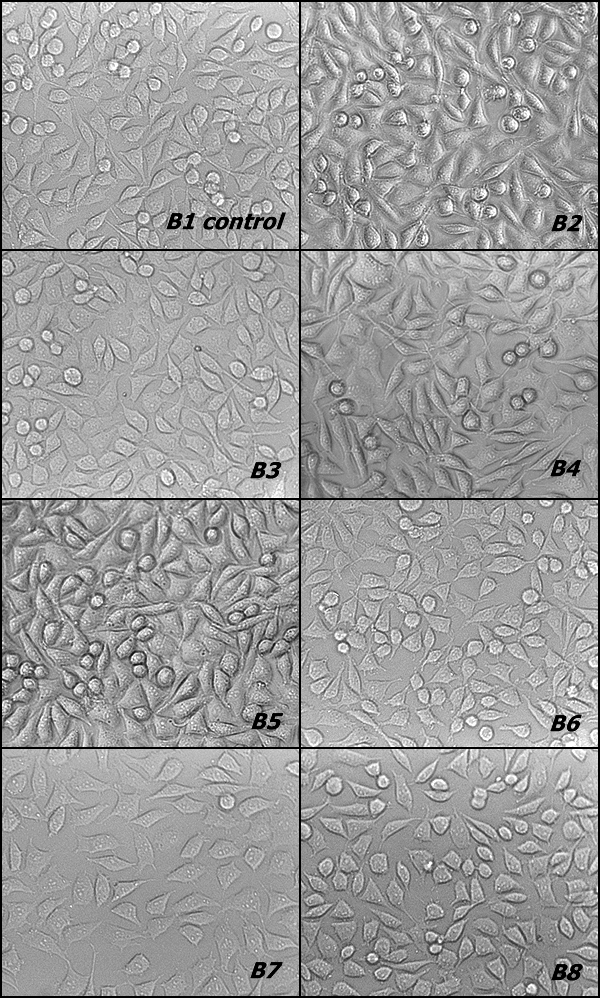 |
| **2h** | **5h** |
| 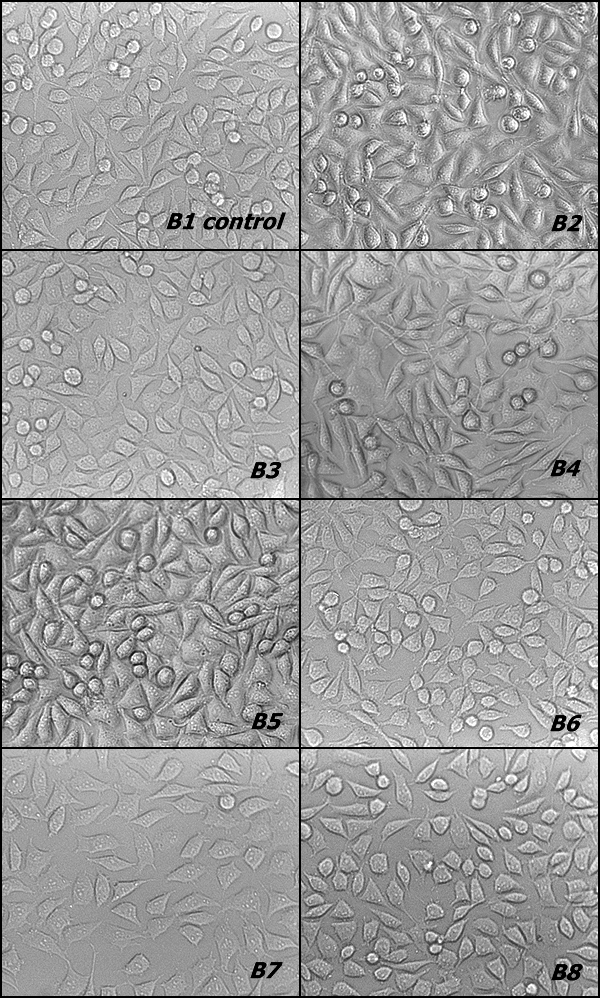 | 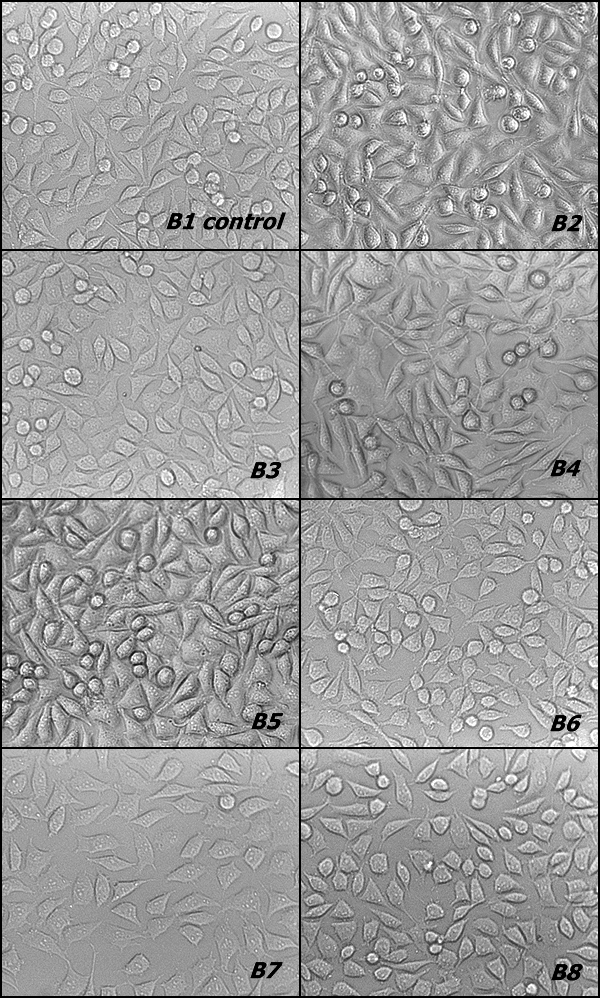 |
| **6b** | **6o** |

**Fig. S1.** Morphology of normal L929 cells and cultured cells with **1f** (50 μg/mL), **1h** (100 μg/mL), **1m** (50 μg/mL), **2h** (50 μg/mL) **5h** (10 μg/mL), **6b** (50 μg/mL), **6o** (5 μg/mL)

**3. SAR parameters of 4-arylthiosemicarbazides 1-6**

Abbreviations of physicochemical parameters collected in Table S1 are as follows: bulk parameter based on molar refraction (*MR*), positionally weighted electronic parameters (*F*) and (*R*), positionally weighted distributive parameter (*π*), lipophilicity (*logP*), surface area (*SA*), volume (*V*), refractivity (*Rf*), polarizabilitiy (*α*), moment dipole (*µ*), the highest occupied molecular orbital energy (*E_HOMO_*), the lowest unoccupied molecular orbital energy (*E_LUMO_*), the difference between HOMO and LUMO energy levels (*HLG*), hardness (*η*) obtained from the equation η = (E_LUMO_ – E_HOMO_)/2, Mulliken electronegativity (*χ*) obtained from the equation χ = -(E_HOMO_ + E_LUMO_)/2, total energy (*E_T_*), binding energy (*E_B_*), isolated atomic energy (*E_IA_*), electronic energy (*E_E_*), core-core interaction (*I_C-C_*), heat of formation (*HF*).

Parameters such as: *MR*, *F*, *R*, *π* were taken from literature. [1] Remaining physicochemical parameters were calculated using HyperChem8.0.3 [2] program at RM1 level of theory.

**Table S1**. SAR parameters^a^ of studied 4-arylthiosemicarbazides

| Comp | MR  F | R  logP | π  SA | V  Rf | α  µ | HOMO  LUMO | HLG  η | χ  E_T_ | E_B_  E_IA_ | E_E_  I_C-C_ | HF  Ref. |
| --- | --- | --- | --- | --- | --- | --- | --- | --- | --- | --- | --- |
| **1a** | -  - | -  2.42 | -  328.39 | 751.36  81.67 | 31.82  3.88 | -8.93  -1.44 | -7.49  3.75 | 5.19  -75544.02 | -2534.37  -73009.65 | -493417.47  417873.45 | 675.90  [3] |
| **1b** | 4.70  -0.07 | -0.12  2.89 | 0.49  350.50 | 793.16  86.71 | 33.65  4.04 | -8.91  -1.43 | -7.48  3.74 | 5.17  -79104.68 | -2783.28  -76321.39 | -541813.27  462708.59 | 702.08  [3] |
| **1c** | 6.00  1.11 | 0.16  0.44 | 0.22  388.93 | 817.84  88.49 | 33.66  3.05 | -9.33  -1.67 | -7.66  3.83 | 5.50  -94561.36 | -2567.62  -91993.74 | -629588.74  535027.38 | 822.67  [3] |
| **1d** | 6.50  0.41 | -0.50  2.17 | -0.12  391.57 | 836.30  88.13 | 34.29  5.30 | -8.95  -1.31 | -7.64  3.82 | 5.13  -86395.69 | -2784.72  -83610.98 | -589644.89  503249.20 | 760.21  [3] |
| **1e** | 4.00  0.79 | 0.16  3.30 | 1.04  358.28 | 818.91  87.64 | 33.38  4.39 | -9.15  -1.62 | -7.53  3.77 | 5.39  -111892.56 | -2993.24  -108899.32 | -728866.77  616974.21 | 392.49  [3] |
| **1f** | 9.60  - | -  3.46 | -  397.76 | 831.13  91.28 | 35.67  2.00 | -9.15  -1.56 | -7.59  3.80 | 5.36  -92594.05 | -2943.49  -89650.56 | -592273.66  499679.61 | 220.55  [3] |
| **1g** | -0.40  0.69 | -0.12  2.56 | 0.22  337.81 | 767.27  81.88 | 31.73  4.47 | -9.08  -1.49 | -7.59  3.80 | 5.29  -86469.88 | -2600.92  -83868.96 | -550565.85  464095.96 | 576.14  [3] |
| **1h** | -0.40  0.71 | -0.34  2.56 | 0.15  345.56 | 770.62  81.88 | 31.73  2.83 | -9.15  -1.40 | -7.75  3.88 | 5.23  -86471.34 | -2602.38  -83868.96 | -546963.43  460492.09 | 574.68  [3] |
| **1i** | -0.80  - | -  2.70 | -  350.93 | 765.86  82.10 | 31.63  2.27 | -9.11  -1.56 | -7.55  3.78 | 5.34  -97391.97 | -2663.70  -94728.27 | -606441.76  509049.79 | 480.14  [3] |
| **1j** | -0.80  - | -  2.70 | -  347.64 | 774.89  82.10 | 31.63  3.83 | -9.12  -1.59 | -7.53  3.77 | 5.36  -97393.21 | -2664.94  -94728.27 | -608541.73  511148.52 | 478.91  [3] |
| **1k** | 7.60  0.73 | -0.18  3.21 | 1.19  374.23 | 816.94  89.29 | 34.44  3.11 | -9.09  -1.52 | -7.57  3.79 | 5.31  -83475.58 | -2604.02  -80871.56 | -539278.50  455802.92 | 580.89  [3] |
| **1l** | 4.80  0.69 | -0.16  2.94 | 0.73  368.45 | 791.75  86.47 | 33.75  2.72 | -9.07  -1.50 | -7.57  3.79 | 5.29  -84069.27 | -2739.16  -81330.11 | -540372.12  456302.85 | 447.99  [3] |
| **1m** | 7.60  0.91 | -0.15  3.21 | 0.84  367.37 | 811.43  89.29 | 34.44  4.09 | -9.01  -1.54 | -7.47  3.74 | 5.28  -83475.46 | -2603.90  -80871.56 | -540850.80  457375.35 | 581.01  [3] |
| **2c** | 6.00  1.11 | 0.16  0.92 | 0.22  395.83 | 804.79  84.80 | 33.24  4.77 | -9.14  -1.21 | -7.93  3.97 | 5.18  -87810.35 | -2365.93  -85444.42 | -571316.25  483505.90 | 969.25  this work |
| **2h** | -0.40  0.71 | -0.34  1.60 | 0.15  351.92 | 752.19  80.79 | 30.58  6.23 | -8.84  -0.90 | -7.94  3.97 | 4.87  -79718.25 | -2398.61  -77319.64 | -491007.47  411289.22 | 723.34  this work |
| **2i** | -0.80  - | -  1.74 | -  358.45 | 760.35  81.01 | 30.49  6.23 | -8.91  -0.97 | -7.94  3.97 | 4.94  -90641.81 | -2462.87  -88178.95 | -548637.31  457995.50 | 625.87  this work |
| **2m** | 7.60  0.91 | -0.15  2.25 | 0.84  374.93 | 800.50  88.20 | 33.30  7.26 | -8.81  -0.89 | -7.92  3.96 | 4.85  -76723.62 | -2401.38  -74322.24 | -485146.02  408422.40 | 728.42  this work |
| **2n** | -0.40  0.88 | -0.29  1.60 | 0.00  343.43 | 751.47  80.79 | 30.58  7.52 | -8.81  -0.90 | -7.91  3.96 | 4.86  -79717.45 | -2397.82  -77319.64 | -494158.98  414441.52 | 724.13  this work |
| **3c** | 6.00  1.11 | 0.16  0.40 | 0.22  376.11 | 788.94  80.60 | 30.99  4.70 | -9.20  -1.30 | -7.90  3.95 | 5.25  -88622.65 | -2669.23  -85953.42 | -580415.93  491793.28 | 764.65  this work |
| **3d** | 6.50  0.41 | -0.50  0.68 | -0.12  378.37 | 805.70  82.84 | 31.49  7.80 | -8.86  -0.43 | -8.43  4.22 | 4.65  -80456.48 | -2885.82  -77570.65 | -538623.75  458167.27 | 702.69  this work |
| **3h** | -0.40  0.71 | -0.34  1.08 | 0.15  332.98 | 737.95  76.59 | 28.93  6.04 | -8.94  -0.61 | -8.33  4.17 | 4.78  -80531.02 | -2702.38  -77828.64 | -499207.97  418676.95 | 518.27  this work |
| **3m** | 7.60  0.91 | -0.15  1.73 | 0.84  353.75 | 781.69  84.00 | 31.65  6.73 | -8.90  -0.59 | -8.31  4.16 | 4.75  -77536.41 | -2705.17  -74831.24 | -494511.22  416974.81 | 523.33  this work |
| **3n** | -0.40  0.88 | -0.29  1.08 | 0.00  338.31 | 740.43  76.59 | 28.93  7.36 | -8.82  -0.38 | -8.44  4.22 | 4.60  -80532.46 | -2703.83  -77828.64 | -500260.42  419727.95 | 516.83  this work |
| **3q** | 4.70  -0.05 | -0.14  1.40 | 0.48  429.98 | 811.48  81.42 | 30.86  3.62 | -8.89  -0.31 | -8.58  4.29 | 4.60  -73171.85 | -2890.78  -70281.07 | -459409.66  386237.82 | 638.18  this work |
| **4b** | 4.70  -0.07 | -0.12  1.52 | 0.49  379.30 | 805.30  83.69 | 31.98  7.15 | -8.76  -0.18 | -8.58  4.29 | 4.49  -78858.25 | -2906.38  -75951.86 | -540463.38  461605.14 | 792.13  this work |
| **4d** | 6.50  0.41 | -0.50  0.80 | -0.12  419.85 | 839.36  85.11 | 32.61  7.28 | -8.76  -0.25 | -8.51  4.23 | 4.51  -86147.40 | -2905.95  -83241.45 | -586604.78  500457.38 | 852.11  this work |
| **4k** | 7.60  0.73 | -0.18  1.84 | 1.19  411.01 | 826.38  86.27 | 32.77  5.06 | -8.96  -0.59 | -8.37  4.19 | 4.78  -83229.10 | -2727.07  -80502.03 | -534898.23  451669.13 | 670.98  this work |
| **4p** | 12.8  0.67 | -0.20  2.31 | 1.43  415.68 | 841.95  91.05 | 35.17  5.63 | -8.83  -0.47 | -8.36  4.18 | 4.65  -80721.98 | -345.64  -80376.35 | -534242.17  453520.18 | 3051.19  this work |
| **4q** | 4.70  -0.05 | -0.14  1.52 | 0.48  404.26 | 813.59  83.69 | 31.98  7.15 | -8.79  -0.33 | -8.46  4.23 | 4.55  -78859.56 | -2907.69  -75951.86 | -534750.38  455890.82 | 790.82  this work |
| **5c** | 6.00  1.11 | 0.16  3.10 | 0.22  434.71 | 918.58  97.43 | 38.26  3.38 | -9.06  -1.24 | -7.82  3.91 | 5.15  -100864.51 | -3240.93  -97623.58 | -723604.30  622739.80 | 980.71  [3] |
| **5h** | -0.40  0.71 | -0.34  3.29 | 0.15  390.51 | 867.61  90.32 | 36.33  5.02 | -8.92  -0.63 | -8.29  4.15 | 4.78  92772.80 | -3274.00  -89498.80 | -633619.74  540846.94 | 734.41  [3] |
| **5i** | -0.80  - | -  3.43 | -  398.37 | 874.24  90.54 | 36.24  5.34 | -8.97  -0.71 | -8.26  4.13 | 4.84  -103696.24 | 3338.13  -100358.11 | -695294.52  591598.28 | 637.07  [3] |
| **5m** | 7.60  0.91 | -0.15  3.94 | 0.84  413.37 | 915.69  97.73 | 39.04  6.43 | -8.87  -0.64 | -8.23  4.12 | 4.76  -89778.19 | -3276.79  -86501.40 | -626521.90  536743.71 | 739.47  [3] |
| **5n** | -0.40  0.88 | -0.29  3.29 | 0.00  389.26 | 876.73  90.32 | 36.33  6.32 | -8.92  -0.65 | -8.27  4.14 | 4.79  -92772.84 | -3274.04  -89498.80 | -634705.81  541932.98 | 734.37  [3] |
| **6b** | 4.70  -0.07 | -0.12  2.13 | 0.49  407.86 | 934.71  104.30 | 38.46  9.47 | -8.66  -1.06 | -7.60  3.80 | 4.86  -88299.61 | -3562.24  -84737.37 | -657712.14  569412.54 | 925.37  this work |
| **6k** | 7.60  0.73 | -0.18  2.46 | 1.19  422.16 | 946.53  106.88 | 39.25  7.87 | -8.80  -1.09 | -7.71  3.86 | 4.94  -92669.57 | -3382.04  -89287.53 | -658998.96  566329.39 | 805.12  this work |
| **6n** | -0.40  0.88 | -0.29  1.80 | 0.00  392.53 | 896.57  99.47 | 36.53  9.89 | -8.72  -1.15 | -7.57  3.79 | 4.94  -95662.93 | -3378.00  -92284.93 | -666319.00  570655.36 | 801.31  this work |
| **6o** | 6.50  0.52 | -0.43  1.41 | -0.13  430.90 | 965.15  105.72 | 39.09  10.50 | -8.52  -1.06 | -7.46  3.73 | 4.79  -95586.68 | -3559.73  -92026.95 | -711623.30  616036.62 | 987.44  this work |
| **6p** | 12.80  0.67 | -0.20  2.92 | 1.43  438.12 | 962.26  111.67 | 41.65  7.94 | -8.77  -1.11 | -7.66  3.83 | 4.94  -90163.50 | -1001.65  -89161.85 | -653586.72  563423.21 | 3184.28  this work |

^a^surfaces and refractivity in Å^2^, volumes and polarizability in Å^3^, moment dipoles in D, energies in kcal/mol

**References**

[1] F.E. Norrington, R.M. Hyde, S.G. Williams, R. Wootton, Physiochemical-activity relations in practice. 1. A rational and self-consistent data bank, J. Med. Chem. 18 (1975) 604-607.

[2] HyperChem 8.0.3, HyperCube Inc., Gainsville, FL, USA, 2007.

[3] A. Siwek, P. Stączek, J. Stefańska, Synthesis and structure-activity relationship studies of 4-arylthiosemicarbazides as topoisomerase IV inhibitors with Gram-positive antibacterial activity. Search for molecular basis of antibacterial activity of thiosemicarbazides, Eur. J. Med. Chem. 46 (2011) 5717-5726.

1. ^^To whom correspondence should be addressed. E-mail: [agata.siwek@am.lublin.pl](mailto:agata.siwek@am.lublin.pl)

   Phone: +48 081 532 05 19. Fax: +48 081 532 45 46. [↑](#footnote-ref-2)
